# Development of the Hausa version of the Pain Catastrophizing Scale: translation, cross-cultural adaptation and psychometric evaluation in mixed urban and rural patients with chronic low back pain

**DOI:** 10.1186/s12955-020-01644-1

**Published:** 2021-02-05

**Authors:** Aminu A. Ibrahim, Mukadas O. Akindele, Bashir Kaka, Naziru B. Mukhtar

**Affiliations:** 1 Department of Physiotherapy, Faculty of Allied Health Sciences, College of Health Sciences, Bayero University Kano, P.M.B 3011, Kano, Kano State Nigeria; 2 Department of Physiotherapy, Muhammad Abdullahi Wase Teaching Hospital, Hospitals Management Board, P.M.B 3160, Kano, Kano State Nigeria

**Keywords:** Chronic low back pain, Cross-cultural adaptation, Hausa, Pain catastrophizing, Pain catastrophizing scale, Reliability, Validity, Translation

## Abstract

**Background:**

Catastrophizing has been recognized as an important contributor to chronicity in individuals with chronic pain syndromes including low back pain (LBP). The Pain Catastrophizing Scale (PCS) is perhaps the most widely used tool to evaluate the degree of pain catastrophizing. However, its use is limited in Hausa-speaking countries due to the lack of a validated translated version.

**Objective:**

To translate and cross-culturally adapt the PCS into Hausa (Hausa-PCS), and evaluate its psychometric properties in mixed urban and rural patients with chronic LBP.

**Methods:**

The PCS was translated and cross-culturally adapted into Hausa in accordance with established guidelines. To evaluate its psychometric properties, a consecutive sample of 200 patients with chronic LBP was recruited from urban and rural Nigerian hospitals. Validity was evaluated by exploring content validity, factorial structure (confirmatory factor analysis [CFA]), construct validity (Spearman’s rho for a priori hypotheses) and known-groups validity. Reliability was evaluated by calculating internal consistency (Cronbach’s α), intraclass correlation coefficient (ICC), standard error of measurement (SEM), minimal detectable change (MDC) and limits of agreement with 95% confidence interval (LOA_95%_).

**Results:**

The Hausa-PCS was comprehensible with good content validity. The CFA confirmed a 3-factor structure similar to the original English version. The concurrent validity was supported as 83% (5/6) of the a priori hypotheses were confirmed. Known-groups comparison showed that the questionnaire was unable to differentiate between male and female or urban and rural patients (*p* > 0.05). Internal consistency and ICC were adequate for the Hausa-PCS total score (α = 0.84; ICC = 0.90) and the subscale helplessness (α = 0.78; ICC = 0.89) but for the subscales rumination (α = 0.69; ICC = 0.68) and magnification (α = 0.41; ICC = 0.43). The LOA_95%_ for the Hausa-PCS total score was between − 8.10 and + 9.75, with SEM and MDC of 3.47 and 9.62 respectively.

**Conclusion:**

The Hausa-PCS was successfully developed and psychometrically adequate in terms of factorial structure, construct validity, internal consistency and test–retest reliability when applied in mixed urban and rural patients with chronic LBP. However, the internal consistency and reliability coefficients (ICC) for the individual subscales are inadequate. Thus, we support the use of the total score when evaluating pain catastrophizing for clinical or research purposes.

## Background

Low back pain (LBP) remains the most common painful musculoskeletal disorder affecting the adult population indiscriminately across the world [1]. It is the greatest contributor to years lived with disability worldwide [2], and imposes a sizable economic, societal and health impact [3, 4]. Though LBP is considered to be a multifactorial disorder associated with numerous possible etiologies and diverse interpretations of the underlying mechanisms [3, 5], in most cases, it is non-specific, signifying that the cause of the pain cannot be reliably identified [6].

While it is commonly believed that most people experiencing a new episode of LBP recuperate within a few weeks, reoccurrences are common and considerable fractions may go on to develop chronic LBP [7]. The development of non-specific chronic LBP is believed to be multifaceted with biomechanical and psychosocial factors being implicated [5, 8]. However, while biomechanical factors appear to have a greater impact on the occurrence of LBP episodes, psychosocial factors seem to have a major impact on its chronicity, as the latter predicts the transition to and maintenance of chronic LBP [6, 8, 9].

One important psychological factor linked with the chronicity of LBP is catastrophizing. According to Sullivan et al. [10], catastrophizing is a maladaptive coping strategy defined as an exaggerated negative mental state related to an actual or anticipated painful experience. Catastrophizing has been recognized as an important mediator to pain behavior and pain-related fear in individuals with chronic pain conditions [11]. It is closely related to fear-avoidance beliefs [12] as the former is thought to be a precursor of the latter [13]. In keeping with the fear-avoidance model, when pain is interpreted as threatening, it influences the use of a catastrophizing pain coping style which in turn may influence pain-related fear to produce avoidance and hypervigilance to bodily sensation that is followed by physical disuse, functional disability, depression and pain chronicity [14]. Plenty of evidence suggests that catastrophizing is a predictor of persistent pain and chronic LBP disability [15–17] as well as a mediator and arbitrator of treatment effectiveness among sufferers of chronic LBP [18]. Thus, evaluating catastrophizing in this group of patients is essential to guide the choice of therapeutic interventions.

The Pain Catastrophizing Scale (PCS) developed by Sullivan et al. [19] in 1995 is perhaps the most widely used tool to evaluate the degree of pain catastrophizing in clinical practice and research. The PCS is a valid and reliable measure of how catastrophizing impact on pain experience [19, 20]. Essentially, it has been proved to be a useful measure of pain catastrophizing in various pain conditions such as chronic LBP [21], chronic neck pain [22], anterior knee pain [23], neuropathic pain [24], postsurgical pain [25], soft tissue injuries [26], respiratory tract illness [27] and dental procedures [28]. Furthermore, the scale has been translated and adapted into many languages/cultures such as the Arabic [29, 30], Afrikaans [31], Brazilian Portuguese [32], Catalans [33], Chinese [34], German [35], Italian [36], Korean [37], Malay [38], Norwegian [39], Simplified Chinese [40], Sinhala [41], Swedish [42], Spanish [43], Turkish [44] and Xhosa [31] versions.

Chronic LBP appears to be a major cause of disability in Nigeria with an estimated annual prevalence of 33–74% [45]. The burden, however, is unduly greater in rural areas compared to urban areas as the one-year prevalence rate of 74% found in rural Nigeria is higher than the 44% found in urban Nigeria [46, 47]. In the same vein, maladaptive beliefs including catastrophizing have been found to be associated with chronic LBP disability in both urban and rural Nigeria [48, 49] similar to that found in western nations [50]. Despite the greatest burden of chronic LBP in Nigeria, self-report outcomes to evaluate cognitive or maladaptive beliefs are generally lacking in the main indigenous Nigerian languages.

There are over 500 native languages spoken in Nigeria, with English being the official language of communication. However, quite a number of patients cannot speak or write in English [51]. The Hausa language is one of the three major native languages spoken in the country particularly in the Northern region. Although the Hausa language is also commonly spoken in many other West African countries [52] with an estimated 50–100 million speakers, the most important dialect is generally regarded as that spoken in Kano, Northwestern Nigeria. This dielect is the standard variety used for official purposes. Therefore adapting the PCS into Hausa in this context will facilitate its use not only in Nigeria but also in other Hausa-speaking countries. The objective of this study was to translate and cross-culturally adapt the PCS into Hausa, and evaluate its psychometric properties in terms of internal consistency, test–retest reliability, and factorial, construct and known-groups validity in mixed urban and rural patients with chronic LBP.

## Methods

### Ethical consideration

This study was approved by the Health Research Ethics Committee, Ministry of Health Kano State (Ref: MOH/Off/797/T.I./651). Written permission (via email) to translate the PCS into Hausa language was obtained from the original developer (Prof. Michael J. Sullivan) and copyright holder (MAPI Research Trust) of the PCS. Written informed consent was obtained from all participants prior to their involvement in the study.

### Study design

Translation, cross-cultural adaptation, test–retest and cross-sectional study of psychometric analysis of the Hausa version of the PCS.

### Outcomes

#### Pain Catastrophizing Scale (PCS)

The PCS consists of 13 items, with each item rated using a 5-point Likert scale ranging from 0 (not at all) to 4 (all the time) [19]. Each item is rated according to respondent’s perceived thoughts and feelings while experiencing pain. The total score ranges from 0 to 52, with higher scores indicating more catastrophic thoughts [19]. The PCS has three dimensions; rumination (4 items), magnification (3 items) and helplessness (6 items). The scale has been shown to have strong construct validity, reliability and stability [19, 20, 53].

#### Visual Analogue Scale for pain (VAS-pain)

The Hausa version of the VAS-pain [54] was used to evaluate levels of the pain intensity. The scale consists of a 100 mm horizontal line anchored on the left with the phrase ‘‘No Pain’’ and on the right with the phrase ‘‘Worst Imaginable Pain”. A higher score indicates greater pain intensity. The respondents were asked to mark a point on the line that best reflects their current pain. The Hausa version of the VAS-pain has adequate alternate forms reliability [54].

#### Oswestry Disability Index (ODI)

The Hausa version of the ODI 2.1a [51] was used to evaluate levels of functional disability. It consists of 10 topics concerning pain intensity, personal care, lifting, walking, sitting, standing, sleeping, sex life, social life and traveling. Each question has six statements scored from 0 to 5. Scores obtained for each topic are summed and divided by the number of answered topics to give a final score out of 100 which indicates the respondent’s percentage perceived level of disability (0–100), with higher scores indicating greater disability. The Hausa version of the ODI 2.1a was found to be a valid and reliable measure of functional disability in chronic LBP patients [51].

#### Fear-Avoidance Beliefs Questionnaire (FABQ)

The Hausa version of the FABQ [55] was used to evaluate fear-avoidance beliefs. It consists of 16 items, with each item scored using a Likert scale ranging from 0 (completely disagree) to 6 (completely agree). The questionnaire consists of two subscales: a 4-item physical activity subscale (FABQ-physical activity) and a 7-item work subscale (FABQ-work). Each subscale scores are summed to give a total score with the FABQ-physical activity subscale having a score ranging from 0 to 24 and the FABQ- work subscale having a score ranging from 0 to 42. Summing the two subscale scores gives a total maximum FABQ score of 66, with higher scores indicating greater fear-avoidance beliefs. The Hausa version of the FABQ is a valid and reliable measure of fear-avoidance beliefs in patients with chronic LBP [55].

#### Short-form Health Survey (SF-12)

The Hausa version of the SF-12 [56] was used to evaluate mental well-being. It consists of 12-item, and evaluates two global health constructs: the physical component summary (PCS-12) and the mental component summary (MCS-12). Each item of the questionnaire has response categories which vary from 2 to 6 and raw scores for items ranging from 1 to 6. To calculate the PCS-12 and MCS-12 sores, a web-based scoring tool (www.orthotoolkit.com/sf-12/) was used. Higher scores indicate better health status. The Hausa version of the SF-12 was shown to be a valid and reliable measure of health-related quality of life in patients with chronic LBP [56].

### Translation and cross-cultural adaptation

The translation procedure was conducted according to the guidelines published by Beaton et al. [57]. The translation included six stages as follows:

1. *Forward translation* The PCS was translated from English into Hausa by two independent bilingual translators (Hausa and English, with Hausa as their first language). The first translator was a clinical physiotherapist and familiar with the concept of the questionnaire. The second translator was a professional translator and unaware of the concept being examined. The translators produced two forward-translated versions (T_1_ and T_2_).
2. *Synthesis of forward translations* The two forward translated versions were then synthesized to one version (T_3_) following consensus between the two forward translators, mediated by the lead author (AAI).
3. *Backward translation* The synthesized version (T_3_) was then back-translated into English by two independent bilingual translators (Hausa and English) who had no medical background and knowledge of the original English version. The translators produced two backward-translated versions (T_4_ and T_5_).
4. *Expert committee review* An expert committee consisting of all forward and backward translators, a methodologist and two of the study authors (AAI and BK) reviewed all the translated versions and reached a consensus on any discrepancy with the aim of achieving semantic, idiomatic, experiential and conceptual equivalences between the original English version and the targeted version. A prefinal version was then produced.
5. *Pilot testing* The prefinal version was tested in a group of 20 patients with chronic LBP recruited from urban and rural Nigerian communities to evaluate comprehensibility and acceptability. Cognitive debriefing was done by the lead author, and problematic items were identified and resolved in consultation with the expert review committee. This stage ensured face and content validity.
6. *Proofreading* A professional translator independently proofread the final version for any minor errors that may have been missed in the previous stages. The final version (see Additional file 1) was then produced and sent to MAPI Research Trust.

### Psychometric evaluation

The procedure used throughout this section has been used in the cross-cultural adaptation of other Hausa self-report measures as described elsewhere [51, 56].

### Sample size estimation

Generally, there is no clear consensus on the required sample size for validation of patient-reported outcome tools [58]. However, “The quality criteria for measurement properties of health status questionnaires” suggest that at least  50 subjects would be adequate for test-retest reliability, construct validity and ceiling/floor effects analyses whereas a minimum of 100 subjects or 4–10 subjects per variable (Rules-of-thumb) would be adequate for internal consistency and factorial structure analyses [58]. Based on these recommendations, 200 participants were recruited to study the psychometric properties of the Hausa version of the PCS (Hausa-PCS).

### Participants and settings

The study was carried out purposely in a selected urban tertiary health facility (Murtala Muhammad Specialist Hospital) and three rural secondary health facilities (Dawakin Kudu General Hospital, Wudil General Hospital and Kura General Hospital), all in Kano State, Northwestern Nigeria. These hospitals were chosen to recruit both urban and rural patients so as to have broader applicability of the questionnaire in these settings. The participants were recruited from the physiotherapy out-patient units of the selected hospitals, from February to May 2018. Eligible participants were those suffering from chronic LBP (defined as having LBP of not less than 12 weeks) between 18 and 70 years old, and fluent in Hausa language. Participants with previous spine surgery, evidence of serious spine pathology for example infection, malignancy, fracture, osteoporosis or ankylosing spondylitis, cognitive or mental impairment were excluded.

### Evaluation of outcomes

Four physiotherapists (with clinical experience between two to five years) were recruited from the selected hospitals and received a one-day training session on the study procedure including interviewer-administration of measures as many Hausa patients especially rural dwellers are non-literate (inability to read and write in Hausa or English). The training was conducted by the primary author. The physiotherapists in each of the selected hospitals were responsible for assessing patients’ eligibility which involves medical history taking, screening of ‘red flags’ (using simple questions about the presence of red flags) to rule out evidence of serious spine pathology, and obtaining patients’ informed consent as well as collecting questionnaire data.

The participants’ socio-demographic information (age, gender, marital status, education level, occupation and habitation) and data on duration of pain, height, weight and body mass index were obtained and documented. The Hausa-PCS along with the Hausa versions of the VAS-pain, ODI, FABQ and SF-12 were administered using interviewer-administration or self-administration method where applicable. The Hausa-PCS was re-administered among 100 participants, 7–14 days after the first administration to assess test–retest reliability.

### Statistical analysis

The normality of the data was tested using visual (normal distribution curve and Q-Q plot) and statistical (Kolmogorov–Smirnov and Shapiro–Wilk’s test) methods. Descriptive statistics of mean, standard deviation (SD), frequencies and percentages were applied to summarize the data. The following statistical approaches were used in evaluating the psychometric properties of the Hausa-PCS.

1. *Content validity:* Content validity refers to the degree to which a scale is relevant and representative of the construct it is designed to measure. Content validity of the Hausa-PCS was evaluated by the expert committee panel during the translation stage. It was also evaluated by examining response trend (using skewness). Items with a skewness > 1.96 suggest a response trend that deviated from a normal distribution pattern [40].
2. *Ceiling and floor effects:* Ceiling or floor effects are considered if more than 15% of respondents scored the maximum or minimum possible score (Table 1). Potential ceiling or floor effects of the Hausa-PCS were investigated by estimating the percentage of respondents indicating the maximum or minimum possible score in all the 13 items of the questionnaire.

**Table 1:** A priori hypotheses for evaluating the psychometric properties of the Hausa Pain Catastrophizing Scale

Psychometric properties	Hypotheses
Floor and ceiling effects	
1. Ceiling effects	15% of the respondents having the maximum score (52) [58]
2. Floor effects	15% of the respondents having the minimum score (0) [58]
Reliability	
1. Internal consistency	Cronbach’s α = 0.70–0.95 [58]
2. Test–retest reliability	Intraclass correlation coefficient = ≥ 0.70 [58]
3. Standard error of measurement	1.6–4.6 [31, 35, 37, 39, 67, 68]
4. Minimal detectable change	4.0–13.0 [31, 35–37, 39, 67, 68]
5. 95% limits of agreement	− 15.1 to + 16.0 [39, 40, 67, 68]
Construct validity	
1. PCS versus FABQ-total, FABQ-physical and FABQ-work	Significant moderate to strong positive correlation (*rho*; 0.34–0.61) [35, 39, 44]
2. PCS versus VAS-pain	Significant moderate to strong positive correlation (*rho*; 0.31–0.64) [36, 37, 39, 67]
3. PCS versus ODI	Significant moderate to strong positive correlation (*rho*; ≥ 0.30) [67]
4. PCS versus MCS-12	Significant moderate to strong negative correlation with (*rho*; ≥ − 0.30) [38]

*PCS* Pain Catastrophizing Scale, *FABQ* Fear-Avoidance Beliefs Questionnaire, *VAS-pain* Visual Analogue Scale for pain, *ODI* Oswestry Disability Index; *rho* Spearman’s correlation coefficient
3. *Factorial validity:* Factorial validity refers to the degree to which the underlying putative structure of a scale is recoverable in a set of test scores. Factor structure of the Hausa-PCS was examined by performing confirmatory factor analysis (CFA) using maximum likelihood estimates. The CFA was carried out with the original three-factor structure (i.e. rumination, magnification and helplessness) extracted by Sullivan et al. [19]. Additionally, the CFA was performed with the one-factor and two-factor structure extracted by Chibnall and Tait [59]. Modification indices were applied to observe for item’s redundancy or those with low factor loadings, and correlation of error terms to improve model fit. The model fit was assessed with four goodness-of-fit indicators including the ratio of chi-square to degrees of freedom (χ^2^/*df*), comparative fit index (CFI), Tucker–Lewis index (TLI), standardized root mean square residual (SRMR) and root mean square error of approximation (RMSEA) [56]. The following cut-off criteria were considered adequate for model fit; χ^2^/*df* of ≤ 2.0, CFI of ≥ 0.95, TLI ≥ 0.90, SRMR ≤ 0.08, and RMSEA ≤ 0.06 [60, 61].
4. *Construct validity:* Construct validity (the degree to which scores of a scale actually measure or test the hypothesis or theory they intended to measure) was evaluated by correlating the Hausa-PCS with measures of pain intensity (VAS-pain), functional disability (ODI), fear-avoidance beliefs (FABQ) and mental well-being (MCS-12). Spearman’s correlation coefficients (*rho*) were used and considered as being strong (*rho* =  > 0.60), moderate (*rho* = 0.30–0.60) and weak/low (*rho* =  < 0.30) [62]. The expected direction and magnitude of the correlations were formulated a *priori* as shown in Table 1. According to Terwee et al. [58], the construct validity is supported when at least 75% (≥ 5) of the predefined hypotheses are confirmed (Table 1).
5. *Known-groups validity:* Known-groups validity (how well an instrument discriminates between relevant known or extreme groups) was evaluated by comparing the Hausa-PCS total score and its subscales with gender and habitant groups using independent *t*-test. We hypothesized that female and rural respondents would have higher pain catastrophizing [63, 64].
6. *Internal consistency:* Internal consistency of the whole questionnaire and its subscales was evaluated with Cronbach’s alpha (α). A Cronbach’s α values of 0.70–0.95 indicate acceptable internal consistency [58].
7. *Test–retest reliability:* Test–retest reliability was assessed by calculating intraclass correlation coefficient (ICC) for agreement using a two-way random effects analysis of variance (ANOVA) model, with a coefficient value ≥ 0.70 indicating acceptable reliability [58]. As per the recommendation of the Consensus-based Standards for the selection of health Measurements Instruments (COSMIN) [65], the standard error of measurement (SEM) and minimal detectable change (MDC) at 95% confidence interval (CI) were calculated to compliment the test–retest reliability. The SEM was computed as the square root of the mean square error term from the reliability ANOVA table. The MDC was then calculated by multiplying the SEM by 2.77 to indicate the minimum amount of change that needs to be observed for it to be considered a true change above measurement error [66]. Additionally, 95% limits of agreement (LOA_95%_) were evaluated with Bland–Altman plots by plotting the difference between baseline and follow-up Hausa-PCS total scores against the mean of Hausa-PCS total scores at baseline and follow-up. A priori hypotheses for the internal consistency (Cronbach’s α), ICC, SEM, MDC and LOA_95%_ for the Hausa-PCS are presented in Table 1.

## Results

### Translation and cross-cultural adaptation

There were no major disagreements between the forward and back translations of the Hausa-PCS. The phrase “I feel I can’t go on” in item 2 was somewhat difficult to translate into Hausa. The translators, however, decided to use the phrase “carry on” in place of “go on” for easy understanding. The translators ensured that standard Hausa was used to attain equivalence between the original English questionnaire and the Hausa version. None of the respondents reported any difficulty with comprehension of the questionnaire items during the pilot testing. Thus, no further modification was carried out and all the items were retained by the expert committee.

### Psychometric testing

#### Socio-demographic and clinical characteristics

Of the 200 participants recruited, the response rate was 100%. There were 123 (61.5%) males and 77 (38.5%) females. Their age ranged between 18–70 years (mean age 45.5 ± 14.5 years). The majority of the participants were living in rural areas (60%). Slightly over half of them were non-literate in Hausa (55.5%) and self-employed (mainly farmers and traders). The socio-demographic and clinical characteristics of the participants are fully presented in Table 2.

**Table 2:** Socio-demographic and clinical characteristics of the participants

Characteristics	N = 200
Age, years, mean ± SD	45.5 ± 14.5
Gender, *n* (%)*,* male: female	123 (61.5), 77 (38.5)
Habitation, *n* (%)*,* urban: rural	80 (40.0), 120 (60.0)
Marital status, *n* (%)*,* married: unmarried	157 (78.5), 43 (21.5)
Educational status, *n* (%)	
Non-formal education	66 (33.0)
Completed primary education	30 (15.0)
Completed secondary education	41 (20.0)
Completed tertiary education	63 (31.5)
Literacy (ability to read and write in Hausa), *n* (%)	
Non-literate	111 (55.5)
Literate	89 (44.5)
Occupational status, *n* (%)	
Paid work (government or private)	49 (24.5)
Self-employed (farming and trading)	112 (56.0)
Student	17 (8.5)
Unemployed	16 (8.0)
Retiree	6 (3.0)
PCS (score range 0–52)	30.0 ± 8.21
FABQ-total (score range 0–66)	36.4 ± 11.4
FABQ-physical activity (score range 0–42)	13.1 ± 5.81
FABQ-work (score range 0–24)	23.3 ± 7.74
VAS-pain (score range 0–100 mm)	36.1 ± 12.6
ODI (score range 0–100)	36.0 ± 10.8

*SD* standard deviation, *PCS* Pain Catastrophizing Scale, *FABQ* Fear-Avoidance Beliefs Questionnaire; *VAS-pain* Visual Analogue Scale for pain, *ODI* Oswestry Disability Index

#### Content validity

The scores for each item of the Hausa-PCS (range: − 0.596 to + 0.573) were normally distributed as none of the item exhibited skewness > 1.96 (Table 3). Thus, none of the items was excluded in the Hausa-PCS.

**Table 3:** General characteristics of the Hausa Pain Catastrophizing Scale (n = 200)

		Range	Mean (SD)	Highest score	Lowest score	Ceiling effects n (%)	Floor effects n (%)	Skewness
Total score		0–52	30.0 (8.21)	52	1	1 (0.5)	3 (1.5)	
Rumination subscale		0–16	10.3 (3.20)	16	4	7 (3.5)	11 (5.5)	
Item 8	I anxiously want the pain to go away	0–4	2.86 (1.10)	4	0	72 (36.0)	0 (0.5)	− 0.596
Item 9	I can’t seem to keep it out of mind	0–4	2.33 (1.09)	4	0	31 (15.0)	2 (1.0)	0.189
Item 10	I keep thinking about how much it hurts	0–4	2.41 (1.07)	4	0	38 (19.0)	1 (0.5)	0.028
Item 11	I keep thinking about how badly I want the pain to stop	0–4	2.81 (1.13)	4	1	76 (38.0)	36 (18.0)	− 0.389
Magnification subscale		0–12	6.99 (2.55)	12	2	6 (3.0)	1 (0.5)	
Item 6	I become afraid that the pain may get worse	0–4	2.26 (1.10)	4	0	31 (15.5)	1 (1.0)	0.106
Item 7	I think of other painful experiences	0–4	2.21 (1.12)	4	0	33 (16.5)	3 (1.5)	0.209
Item 13	I wonder whether something serious may happen	0–4	2.51 (1.17)	4	1	54 (27.0)	59 (29.5)	− 0.071
Helplessness subscale		0–24	12.7 (4.20)	24	2	1 (0.5)	1 (0.5)	
Item 1	I worry all the time whether the pain will end	0–4	2.40 (1.04)	4	1	37 (18.5)	47 (23.5)	0.121
Item 2	I feel I can’t go on	0–4	1.96 (0.96)	4	0	14 (7.0)	3 (1.5)	0.458
Item 3	It’s terrible and I think it’s never going to get any better	0–4	2.21 (1.08)	4	1	31 (15.5)	1 (0.5)	0.271
Item 4	It’s awful and I feel that it overwhelms me	0–4	1.92 (1.07)	4	0	22 (11.0)	5 (2.5)	0.573
Item 5	I feel I can’t stand it any more	0–4	2.15 (1.07)	4	0	29 (14.5)	1 (0.5)	0.372
Item 12	There is nothing I can do to reduce the intensity of the pain	0–4	2.08 (1.06)	4	0	24 (12.0)	1 (0.5)	0.415

*SD* standard deviation

#### Ceiling and floor effects

All the respondents completed the Hausa-PCS without missing values. Ceiling effects were found for items 1, 3, 6, 7, 8, 10, 11 and 13 whereas floor effects were found for items 1, 11 and 13. No ceiling or floor effects were seen in the Hausa-PCS total score or subscales (Table 3).

#### Factorial structure

**Fig. 1:**
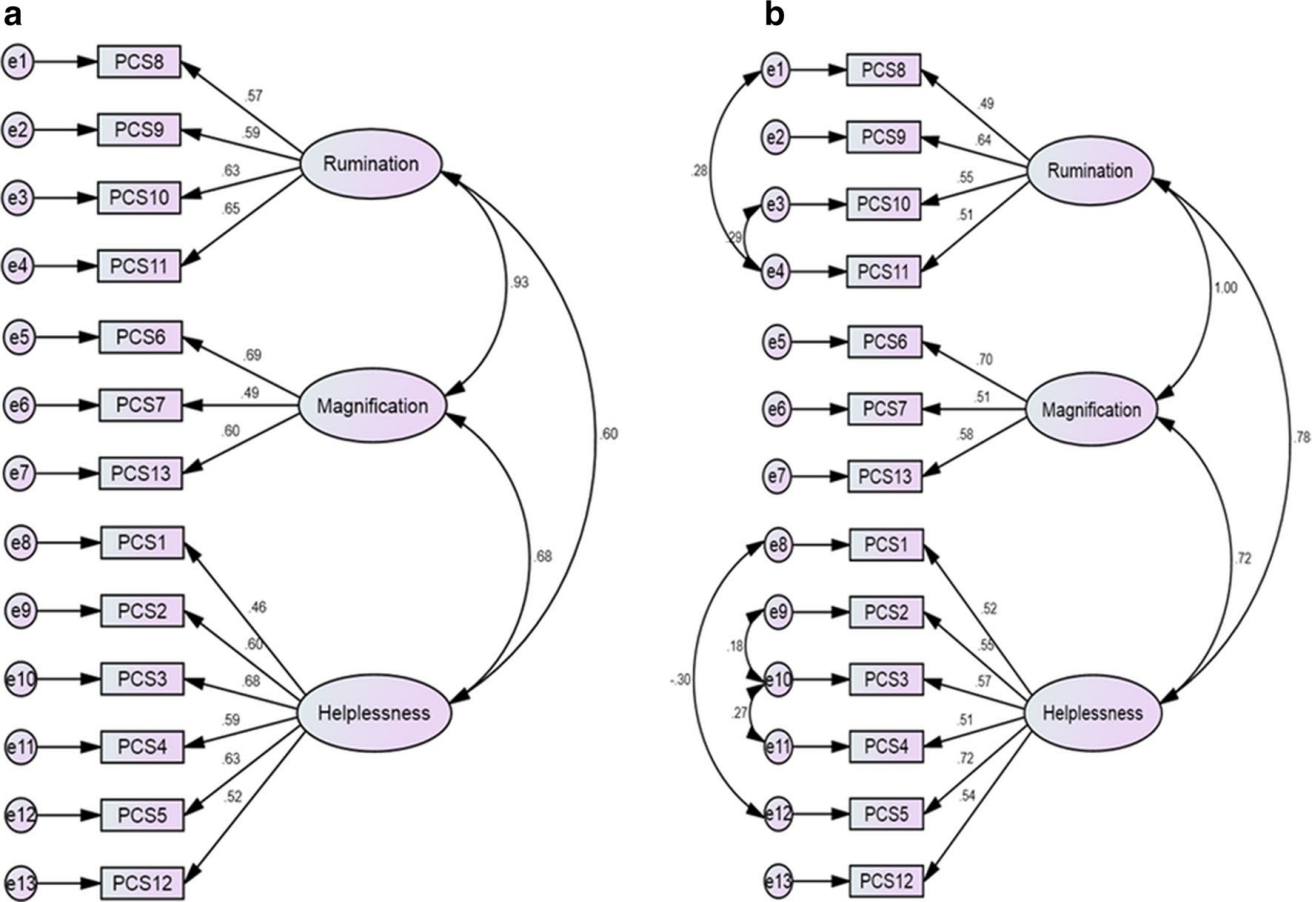
Factor structure of the Hausa Pain Catastrophizing Scale three-factor model. **a** Model without modifications. **b** Model with modifications. (n = 200)

Table 4 shows the results of the CFA for the one-factor, two-factor and three-factor structures of the Hausa-PCS with and without modifications. All the tested models demonstrated poor fit as indicated by the fit indices except the three-factor structure after modifications. Modifications of the three-factor structure were done by allowing 5 error terms to covary (e1–e4, e3–e4, e8–e12, e9–e10 and e10–e11) (Fig. 1).

**Table 4:** Confirmatory factor analyses of the Hausa Pain Catastrophizing Scale models (n = 200)

Model and modifications	Confirmatory factor analysis					
	χ^2^ (*df*)	χ^2^/*df*	CFI	TLI	SRMR	RMSEA (95%CI)
1. One-factor structure						
(a) No modifications	194.9 (65)	3.00	0.796	0.755	0.083	0.100 (0.084–0.117)
(b) With modifications	116.5 (60)	1.94	0.911	0.884	0.065	0.069 (0.050–0.087)
2. Two-factor structure						
(a) No modifications	166.9 (64)	2.60	0.838	0.803	0.080	0.090 (0.073–0.107)
(b) With modifications	111.5 (60)	1.85	0.919	0.895	0.066	0.066 (0.046–0.084)
3. Three-factor structure						
(a) No modifications	132.4 (62)	2.13	0.889	0.861	0.724	0.076 (0.058–0.093)
(b) With modifications	86.8 (57)	1.52	0.953	0.936	0.056	0.051 (0.028–0.072)

*χ*^2^ chi-square, *df* degrees of freedom, *CFI* comparative fit index, *TLI* Tucker–Lewis index, *SRMR* standardized root mean square residual, *RMSEA* root mean square error of approximation, *CI* confidence interval

#### Construct validity

The Hausa-PCS total score demonstrated a strong positive correlation with VAS-pain (*rho* = 0.74, *p* < 0.001) and a moderate positive correlation with FABQ-total (*rho* = 0.42, *p* < 0.001), FABQ-physical activity (*rho* = 0.32, *p* < 0.001), FABQ-work (*rho* = 0.36, *p* < 0.001) and ODI (*rho* = 0.35, *p* < 0.001) as hypothesized (Table 1). However, the correlation between the Hausa-PCS and MCS-12 was weakly negative (*rho* = − 0.20, *p* < 0.05) (Table 5). Overall, 83% (5/6) of the a *priori* hypotheses were confirmed (Table 1).

**Table 5:** Construct validity of the Hausa Pain Catastrophizing Scale (n = 200)

Measures	Pain Catastrophizing Scale		
	*rho*	*P* value	Hypothesis confirmed (Yes/no)
FABQ-total	0.42	0.000	Yes
FABQ-physical activity	0.32	0.000	Yes
FABQ-work	0.36	0.000	Yes
Visual Analogue Scale for pain	0.74	0.000	Yes
Oswestry Disability Index	0.35	0.000	Yes
MCS-12	− 0.20	0.004	No

All outcomes are in Hausa. *FABQ* Fear-Avoidance Beliefs Questionnaire, *rho* Spearman’s correlation coefficient, *MCS-12* mental component summary

#### Known-groups validity

Known-groups comparison of the Hausa-PCS with regard to gender and habitation groups showed no significant differences in the questionnaire total score and its subscales (*p* > 0.05) (Table 6).

**Table 6:** Known-groups comparison of the Hausa Pain Catastrophizing Scale

	Gender				Habitation			
	Male	Female	t-cal	*p*-value	Urban	Rural	t-cal	*p*-value
	Mean (SD)	Mean (SD)			Mean (SD)	Mean (SD)		
Total score	30.2 (8.29)	29.8 (8.14)	2.559	0.056	30.2 (7.96)	29.9 (8.41)	0.106	0.745
Rumination	10.5 (3.26)	9.34 (3.53)	2.453	0.068	9.93 (3.57)	10.1 (3.29)	0.126	0.723
Magnification	7.30 (2.56)	6.15 (2.42)	2.593	0.057	6.62 (2.24)	7.05 (2.78)	0.672	0.414
Helplessness	13.1 (4.51)	12.4 (4.47)	1.550	0.207	12.7 (4.04)	13.0 (4.82)	0.065	0.799

*SD* standard deviation

#### Internal consistency

As shown in Table 7, the internal consistency as measured by the Cronbach’s α, if item deleted was high (0.837) for the Hausa-PCS total score. Also, adequate internal consistency was obtained for the subscale helplessness (α = 0.78) but for the subscales rumination (α = 0.69) and magnification (α = 0.41) (Table 7).

**Table 7:** Internal consistency and test–retest reliability of the Hausa Pain Catastrophizing Scale

	Internal consistency (n = 200)	Test–retest reliability (n = 100)					SEM	MDC
	Cronbach’s α	Mean (SD) test	Mean (SD) retest	t1–t2	*p* value for test–retest	ICC (95% CI)		
Total (0–52)	0.84	29.8 (8.46)	28.5 (8.19)	1.25	0.012*	0.90 (0.85–0.93)	3.47	9.62
Rumination (0–16)	0.69	10.0 (3.40)	9.40 (3.11)	0.67	0.028*	0.68 (0.52–0.78)	2.24	6.20
Magnification (0–12)	0.41	6.87 (2.56)	7.05 (3.54)	− 0.18	0.630	0.43 (0.16–0.62)	2.63	7.29
Helplessness (0–24)	0.78	12.9 (4.48)	12.1 (4.17)	0.76	0.005*	0.89 (0.83–0.93)	1.88	5.21

*SD* standard deviation, *t1–t2* mean values at test subtracted from retest, *ICC* intraclass correlation coefficient, *CI* confidence interval, *SEM* standard error of measurement, *MDC* minimal detectable change

*P < 0.05

#### Test–retest reliability

**Fig. 2:**
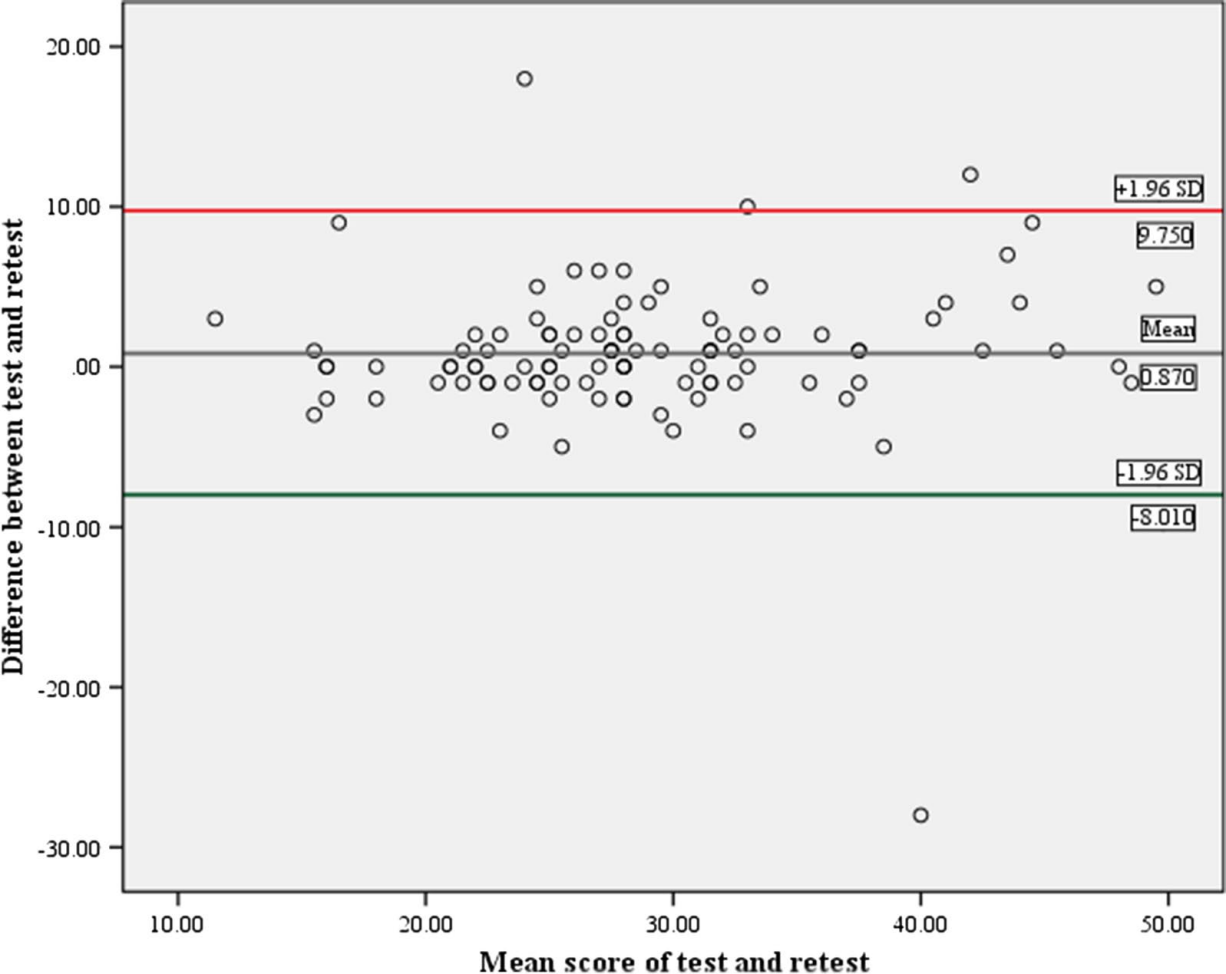
Test–retest agreement of the Hausa Pain Catastrophizing Scale using Bland–Altman procedure (n = 100). Note: Y-axis represents the change in Hausa-PCS scores between baseline and follow-up measurements and X-axis represents the mean of the Hausa-PCS scores at the baseline and follow-up measurements. The center line is the mean change of score (0.870); upper (9.750) and lower (8.010) lines are the limits of agreement for 95% confidence intervals

The ICC for the Hausa-PCS total score was good (0.90; CI: 0.85–0.93). Similarly, acceptable ICC was obtained for the helplessness subscale (0.89; CI: 0.83–0.93) but for the subscales rumination (0.68; CI: 0.52–0.78) and magnification (0.43; CI: 0.16–0.62) (Table 7). The SEM for the Hausa-PCS total score and its subscales are presented in Table 7. The Bland–Altman analysis showed a mean difference between test and retest of 0.87, with LOA_95%_ of − 8.10 to + 9.75 (Fig. 2).

## Discussion

To enable easy assessment of pain catastrophizing and design appropriate interventions targeting this psychological construct in Hausa-speaking patients with LBP, this study described the development of the Hausa-PCS through translation and cross-cultural adaptation of the PCS into Hausa, and finally validation of the translated version in mixed urban and rural individuals with chronic LBP. The results of the study suggested that the Hausa-PCS was comprehensible, valid and reliable when evaluating catastrophic thinking related to pain in Hausa-speaking patients with chronic LBP.

The PCS was fairly simple to translate as there were no serious translation issues encountered. The items of the questionnaire were comprehensible during the field verbal pretesting with urban and rural participants. The translators ensured that standard Hausa wordings and phrases were used for easy understanding in both urban and rural contexts with the goal of achieving conceptual equivalence rather than literal translation. Although no ceiling or floor effects were observed in the total score or the subscales similar to reports of previous studies [39, 69], however, ceiling effects were seen in 8 out of the 13 items whereas floor effects were seen in only 3 items. In line with our findings, ceiling effects in more than half of the PCS items were also reported in the Norwegian validation [39]. In contrast, respondents exhibiting floor and ceiling effects were removed in the validation of the Simplified Chinese PCS among chronic pain patients [40].

The mean total score of the Hausa-PCS was 30.0 comparable to both the urban (30.2) and rural (29.9) respondents, indicating that the studied population experienced a high level of pain catastrophizing considering the report that pretreatment score of greater than 24 was associated with high follow-up pain outcomes [70]. Thus, it can be inferred that individuals with pain catastrophizing scores greater than 24 as in the case of our sample may warrant interventions targeting to reduce pain catastrophizing. Similar to the Simplified Chinese version of the PCS [40], the content validity of the Hausa-PCS in terms of skewness was acceptable as all the items were less than 1.96, suggesting a response trend for normally distributed scores.

The PCS has been widely reported as three-factor structure consisting of the rumination, magnification and helplessness subscales following exploratory factor analysis [20, 33, 35–38, 40, 41, 43, 59, 67] even though minor differences exist regarding how the PCS items loaded onto factors. A two-factor structure has been also reported in the literature [20, 44, 68, 71–73]. In the present study, the CFA suggests that the three-factor structure proposed by Sullivan et al. [19] had the best fit for our sample compared to the one-factor or two-factor structure proposed by Chibnall and Tait [59] as indicated by the low SRMR and RMSEA as well as high CFI and TLI values. These findings correspond with the reports of many validations conducted among patients with diverse chronic pain conditions [35, 71–73]. On the contrary, other validations found the two-factor structure of the PCS to exhibit adequate model fit [44, 68]. In another vein, Huijer et al. [30] found the one-factor, two-factor (based on the authors’ exploratory factor analysis) and Sullivan’s original three-factor structures to exhibit adequate fit to Arabic population. However, it is important to note that the differences in the factor structure of the PCS across studies may be attributed to cultural differences in different countries.

According to the recommendations of the quality criteria for measurement properties of health status questionnaires [58], construct validity is supported when at least 75% of the predefined hypotheses are verified. Based on our a *priori* hypotheses that the Hausa-PCS total scores would correlate moderately to strongly with the criterion variables, the construct validity was supported as 83% (5 out of 6) of the hypotheses were confirmed. The questionnaire demonstrated a strong positive correlation with the VAS-pain (*rho* = 0.74) comparable to that obtained for the Hindi version (*rho* = 0.65) [67] and higher than that (*rho* range = 0.19–0.52) reported by many other adapted versions [29, 32, 35–37, 39, 40, 73]. The moderate positive correlation coefficients obtained with the FABQ-physical activity (*rho* = 0.32) and FABQ-work (*rho* = 0.36) subscales were smaller compared to that obtained for the German (FABQ-physical activity; *rho* = 0.51 and FABQ-work; *rho* = 0.61) [35] and Turkish (FABQ-physical activity; *rho* = 0.49 and FABQ-work; *rho* = 0.47) [44] versions but comparable to the Norwegian version (FABQ-physical activity; *rho* = 0.34 and FABQ-work; *rho* = 0.25) [39] except for the FABQ-work subscale which was found to be very low in the later version. Similarly, the moderate positive correlation coefficient obtained between our questionnaire and the ODI (*rho* = 0.35) coincides with the 0.35 obtained in the Hindi version [67] but slightly lower than the range of 0.40–0.57 obtained by other versions using the Roland-Morris Disability Questionnaire [35, 36, 69]. In another vein, the Hausa-PCS had a weak negative correlation coefficient with the MCS-12 scores (*rho* = − 0.20) contrary to the Malay version which demonstrated a moderate negative correlation with the MCS-12 scores (*rho* = − 0.38) [38]. The variation in correlation coefficients across studies could be explained for the variation in the studied populations besides the different questionnaires used, for example, Roland-Morris Disability Questionnaire in place of the ODI.

The result of the known-groups validity of the Hausa-PCS revealed that the questionnaire and its subscales are not influenced by socio-demographic variables in terms of gender and habitation. Although this aspect of validity may require further investigation, it can be deducted based on the studied population that male and female as well as urban and rural patients are likely to experience the same level of pain catastrophizing as a result of chronic LBP. In contrast, the Persian version demonstrates its ability to differentiate male and female patients with non-malignant musculoskeletal pain [64].

Regarding internal consistency, the Hausa-PCS total score exhibited adequate internal consistency (α = 0.84) consistent with the original English version (α = 0.87) [74] and the range of 0.84–0.93 reported by many validation studies [32–34, 36–39, 41]. However, we obtained lower alpha coefficients for the rumination (α = 0.69) and magnification (α = 0.41) subscales but sufficient for the helplessness subscale (α = 0.78). Consistent with our findings, most previous studies [33, 36, 39, 67, 73] found lower alpha coefficients for the magnification subscales, which could be attributed to the small number of items peculiar with the three-factor structure. It is important to note that increasing the number of scale items typically increases the Cronbach's alpha [75]. Thus, caution should be exercised when considering the magnification as independent subscale in computing pain catastrophizing. Consequently, the two-factor structure of the PCS may be considered but may warrant further investigation.

The test–retest reliability of the Hausa-PCS total score was highly adequate (ICC = 0.90), suggesting excellent reproducibility. Our value is higher than the original English version (ICC = 0.73) [19] and the range of 0.76–0.85 obtained by several language versions [29, 33, 35–39, 41, 43], consistent with the 0.90 obtained for the Afrikaans [31], Nepali [68] and Xhosa [31] versions but slightly lower than the range of 0.92–0.97 obtained by other language versions [32, 34, 40, 67]. However, for the Hausa-PCS subscales, the ICC was only adequate for the helplessness subscale (ICC = 0.89). The magnification (ICC = 0.68) and rumination (ICC = 0.43) subscales had insufficient test–retest reliability which is consistent with the findings of previous validations demonstrating smaller ICC values for these subscales compared to the helplessness subscale [35–37]. These findings, thus, suggest that further investigation into the factorial structure of the Hausa-PCS may be useful.

The SEM and MDC at 95% CI were computed in this study to supplement the test–retest reliability since ICC does not account for the size of measurement error that is clinically meaningful [56]. The smaller the SEM the better the reliability (precision) of the measure whereas the smaller the MDC the more sensitive is the measure [76]. In the present study, the SEM (3.47) and MDC (9.62) values calculated for the Hausa-PCS total score were comparable to the values calculated for the Afrikaans (SEM = 3.30; MDC = 9.00) [31] and Xhosa (SEM = 3.30; MDC = 9.30) [31] versions; lower than the values calculated for the Korean (SEM = 3.72; MDC = 10.3) [37], German (SEM = 4.60; MDC = 12.8) [35] or Norwegian (SEM = 4.60; MDC = 12.8) [39] versions; but higher than the values calculated for the Hindi (SEM = 1.90; MDC = 5.26) [67] and Nepali (SEM = 2.52; MDC = 6.98) [68] versions. Compared to the SEM and MDC values of the Hausa-PCS total score, the three subscales of the questionnaire demonstrated lower values consistent with the reports of prior studies [31, 39, 67]. Regarding our SEM for the Hausa-PCS total score (3.47), it can be interpreted that if an individual has a baseline total score of 29.0, we can be 95% confident that the true score lies between 25.5 and 32.5. As for the MDC (9.62), a change of 9.63 or above can be considered as a true change in the total score above measurement error. Additionally, the result of the Bland–Altman plot for the Hausa-PCS total score showed minimal bias as the mean difference (0.87) calculated was close to zero, with LOA_95%_ of − 8.10 to 9.75 which lies within the range of − 15.1 to 16.0 reported in the literature [39, 40, 67, 68].

One strength of this study is that the translation and cross-cultural adaptation process were conducted as per the recommendation of guidelines outlined by Beaton et al. [57]. Additionally, the psychometric evaluation was conducted and reported in line with the COSMIN guidelines [65] even though we did not use the global rating of change scale to confirm the respondents’ stability when assessing the test-retest reliability. However, one potential limitation of this study is that the correlations of the Hausa-PCS with the criterion variables used were based on cross-sectional data. Thus, any causal conclusion concerning the influence of pain catastrophizing on pain intensity, functional disability, fear-avoidance beliefs and mental health could not be drawn. Another potential limitation is that we were unable to evaluate responsiveness. Future research is needed to examine the causal relationships between the Hausa-PCS and the aforementioned criterion measures in similar populations. Moreover, further research to evaluate responsiveness in order to establish minimum important change would be useful.

## Conclusion

The Hausa-PCS was successfully developed and psychometrically adequate in terms of factorial structure, construct validity, internal consistency and test–retest reliability when applied in mixed urban and rural patients with chronic LBP. However, the internal consistency and reliability coefficients (ICC) for the individual subscales are inadequate, thus warranting further investigation. The tool can be used especially when considering the total score to evaluate pain catastrophizing for clinical or research purposes.

## Supplementary Information

**Additional file 1:** The Hausa version of the Pain Catastrophizing Scale.

## Data Availability

The data that support the findings of this study are available from the corresponding author upon reasonable request.

## Abbreviations

LBP: Low back pain
PCS: Pain Catastrophizing Scale
VAS-pain: Visual Analogue Scale for pain
ODI: Oswestry Disability Index
FABQ: Fear-Avoidance Beliefs Questionnaire
SF-12: Short-form Health Survey
PCS-12: Physical component summary
MCS-12: Mental component summary
SD: Standard deviation
CFA: Confirmatory factor analysis
CFI: Comparative fit index
TLI: Tucker–Lewis index
SRMR: Standardized root mean square residual
RMSEA: Root mean square error of approximation
ICC: Intraclass correlation coefficient
ANOVA: Analysis of variance
COSMIN: Consensus-based Standards for the selection of health Measurements Instruments
SEM: Standard error of measurement
MDC: Minimal detectable change
CI: Confidence interval
LOA: Limits of agreement
