# Supplementary material for: Development of the Hausa version of the Pain Catastrophizing Scale: translation, cross-cultural adaptation and psychometric evaluation in mixed urban and rural patients with chronic low back pain
Source: Health Qual Life Outcomes. 2021 Feb 5;19:44. doi: 10.1186/s12955-020-01644-1 (PMC7863472; doi:10.1186/s12955-020-01644-1)
Supplement: Supplementary file 1 — Additional file 1: The Hausa version of the Pain Catastrophizing Scale. [file 12955_2020_1644_MOESM1_ESM.pdf]

PAIN CATASTROPHIZING SCALE
(HAUSA VERSION)

Kowane mutum yana samun kansa cikin wani hali maras daɗi a wani lokaci a rayuwarsa. Irin wannan yanayi na iya kasancewa ciwon kai ko na haƙori ko na gaɓɓai ko na na jiki. Mutane suna cin karo da wasu yanaye-yanaye da za su iya jawo musu ciwo kamar rashin lafiya ko jin rauni ko ciwon haƙori ko kuma yin tiyata.

Abin da muke da sha'awar sani shi ne, irin tunani ko abin da ka/ki ke ji idan kana fama da raɗaɗin ciwo. Ga wasu jerin bayanai nan a ƙasa guda (13) da ke nuna irin tunane-tunane da yadda ake ji waɗanda za a iya alaƙantawa da jin raɗaɗin ciwo. Ta amfani da wannan a matsayin ma'auni, ana so ka/ki nuna matsayin yadda ka/ki ke jin wannan tunanin ko jin wani abu a yayin da ka/ki ke cikin raɗaɗin ciwo.

0 - Ba na ji sam **1-** Ina ji kaɗan **2 -** Tsaka-tsaki **3 -** Ina ji sosai **4 -** Koyaushe

A yayin da nake cikin raɗaɗin ciwo…

1	☐	Koyaushe nakan damu cewar ko raɗaɗin zai daina.
2	☐	Na kan ji ba zan iya cigaba ba.
3	☐	Abin yakan yi tsanani, harta kai na fara tunanin ba zai yi sauƙi ba.
4	☐	Na kan firgita kuma na ji abin ya sha kaina.
5	☐	Na kan ji ba zan iya cigaba da jurewa ba.
6	☐	Na kan firgita kan cewa zafin ciwon zai ta'azzara.
7	☐	Na kan shiga tunanin wasu yanaye-yanaye na ciwo.
8	☐	Na kan ƙagu da raɗaɗin ciwon ya daina.
9	☐	Na kan ji cewa bazan iya fitar da abin daga raina ba.
10	☐	Na kan yi tunanin irin raɗaɗin da nake ji.
11	☐	Na kan yi ta tunanin irin yadda nake matuƙar son raɗaɗin ya daina.
12	☐	Babu wani abu da zan iya yi don rage kaifin ciwon.
13	☐	Ina tsoron ko wani abin da ya fi wannan na iya faruwa.
